# The effect of olive leaf extract on cardiovascular health markers: a randomized placebo-controlled clinical trial

**DOI:** 10.1007/s00394-020-02397-9

**Published:** 2020-10-09

**Authors:** Yala Stevens, Bjorn Winkens, Daisy Jonkers, Adrian Masclee

**Affiliations:** 1 Department of Internal Medicine, Division of Gastroenterology-Hepatology, School of Nutrition and Translational Research in Metabolism (NUTRIM), Maastricht University, P. O. Box 616, 6200 Maastricht, The Netherlands; 2 BioActor BV, Maastricht, The Netherlands; 3 Department of Methodology and Statistics, Care and Public Health Research Institute (CAPHRI), Maastricht University, Maastricht, The Netherlands

**Keywords:** Olive leaf extract, Overweight, Cardiovascular disease, Blood lipid profiles

## Abstract

**Purpose:**

Overweight and obesity are associated with many health problems, including cardiovascular disease (CVD). Evidence from previous studies has shown that extracts from olive leaves rich in olive phenolics are able to positively affect CVD risk factors, such as high blood pressure and dyslipidemia. The aim of this study was to investigate the effect of 8-week olive leaf extract (OLE) administration on blood lipid profiles in overweight/obese subjects with mildly elevated cholesterol levels.

**Methods:**

In this randomized, double-blind, placebo-controlled study, 77 healthy adult overweight/obese subjects (aged 56 ± 10 years and BMI 29.0 ± 2.7 kg/m^2^) with total cholesterol levels of 5.0–8.0 mmol/L (5.9 ± 0.7 mmol/L) were randomly assigned to receive 500 mg of OLE (*n* = 39) or placebo (*n* = 38) for 8 weeks. In total, 74 subjects completed the entire study protocol. At baseline, after 4 weeks, and after 8 weeks of supplementation, blood lipid profiles, oxidized low-density lipoprotein (oxLDL), blood pressure, glucose, and insulin levels were assessed. In addition, liver function parameters were measured at baseline and after 8 weeks.

**Results:**

OLE supplementation did not significantly affect blood lipid levels after 4 weeks or after 8 weeks compared to placebo (all *p* > 0.05). For oxLDL, blood pressure, glucose, and insulin levels and liver function parameters, also no statistically significant differences were found between the two intervention groups (all *p* > 0.05).

**Conclusions:**

Blood lipid profiles were not significantly affected by 8 weeks OLE supplementation in overweight/obese subjects with mildly elevated cholesterol levels.

**Trial registered:**

The trial has been registered at ClinicalTrials.gov (NCT02990637).

**Electronic supplementary material:**

The online version of this article (10.1007/s00394-020-02397-9) contains supplementary material, which is available to authorized users.

## Introduction

The prevalence of overweight and obesity has increased rapidly over the last decades and poses a major public health concern, as excess body weight is associated with many health problems such as diabetes, liver disease, cardiovascular disease (CVD), and cancer [1, 2]. Atherosclerosis is an important precursor for CVD. It is considered to start with damage to the endothelial layer caused by CVD risk factors associated with obesity, such as high blood pressure and dyslipidemia [3, 4]. The latter includes increased blood cholesterol levels, which is also widely used as screening marker in clinical practice. In case of markedly elevated cholesterol levels (total cholesterol > 8.0 mmol/L), treatment with cholesterol-lowering medication is usually prescribed. For individuals with moderately or slightly elevated cholesterol levels, instead of medication, the use of lifestyle interventions such as dietary changes or dietary supplements is recommended [5].

Evidence from epidemiological studies and randomized-controlled trials has shown that the Mediterranean diet is associated with a significantly lower incidence of CVD [6]. One of the main components of this diet, olive oil, is assumed to be, at least in part, responsible for a decreased CVD risk [7–9]. Although this effect could be due to various different constituents of olive oil, several studies have shown favorable effects on CVD risk biomarkers resulting from the intake of phenolic compounds present in olives and in olive oil [10–13]. The main phenolics present in the olive plant and olive oil are oleuropein, tyrosol, and hydroxytyrosol. Olive leaves contain the highest overall concentration of these compounds, especially oleuropein, compared to other parts of the plant [13].

Previous studies investigating the effects of extracts from olive leaves rich in olive phenolics have shown that these extracts are able to positively affect CVD risk factors [14–27]. Although much of the evidence has been obtained from animal and in vitro studies [14–22], human data are also available [23–27]. Human intervention studies in subjects with elevated blood pressure, diabetes, overweight, or osteopenia indicate that olive leaf extract (OLE) supplementation favorably affects outcomes such as blood pressure, glucose metabolism, and blood lipid profiles [20, 23–27]. Although these results are promising, the effects of OLE on blood lipid profiles were not the primary aim in any of these studies, nor were participants selected based on their cholesterol levels. Therefore, the primary objective of our study was to investigate the effect of 8-week OLE administration on blood lipid profiles in overweight/obese subjects with mildly elevated cholesterol levels. The secondary objective was to investigate the effect of OLE on lipid peroxidation and blood pressure. Additionally, effects on parameters of glucose metabolism and liver function were assessed. We hypothesize that supplementation with OLE for 8 weeks will improve blood lipid profiles, lipid peroxidation, blood pressure, glucose metabolism, and liver function.

## Methods

The study has been approved by the Medical Ethics Committee of Maastricht University Medical Center + (MUMC +), Maastricht, The Netherlands, and was conducted in full accordance with the Declaration of Helsinki (as amended by the World Medic Association in 2013) and the Dutch Regulations on Medical Research Involving Human Subjects (WMO 1998). All subjects gave their written informed consent before participation. This study has been registered at ClinicalTrials.gov (NCT02990637).

### Subjects

Healthy subjects, aged 18–70 years with a body mass index (BMI) between 25 and 35 kg/m^2^ and elevated cholesterol levels, were recruited by means of advertisements in local newspapers and posters on notice boards at the Maastricht University buildings and from an existing cohort of eligible subjects. For this study, we sought participants with baseline total cholesterol levels in the range of 5.0–8.0 mmol/L, based on the guidelines of the Dutch heart association (https://www.hartstichting.nl/). Cholesterol values within this range are classified as elevated, while values above 8.0 mmol/L are classified as markedly elevated and generally result in the prescription of cholesterol-lowering mediation. Exclusion criteria were: (history of) chronic or severe diseases that may affect study outcomes or limit participation in the study; use of medication influencing endpoints of the study; administration of investigational drugs or participation in any scientific intervention study which could interfere with the study; use of antibiotics within 30 days prior to the start of the study; use of antioxidants, minerals and vitamin supplements; pregnancy or lactation; abuse of alcohol (> 20 alcoholic units/week) or recreational drugs; smoking; recent weight gain or loss (> 3 kg in previous 3 months); high physical activity (> 4.5 h of running/week); history of any side effects towards intake of olives. In total, 109 subjects were assessed for eligibility. Of these, 77 subjects were included in the current study (Supplemental Fig. 1).

### Study design

The study was designed as a randomized, parallel, double-blind, placebo-controlled trial, conducted at the Metabolic Research Unit of Maastricht University, Maastricht, The Netherlands. The study participants were randomly assigned to receive one of the following two interventions: placebo or OLE. The randomization list was computer-generated by an independent person, with random and concealed block sizes of 2, 4, and 8 for treatment allocation. Participants and researchers were blinded to the intervention allocations until all analyses were completed. The total treatment duration was 8 weeks and measurements were performed at three different time points, *i.e.,* at baseline (test day 1), after 4 weeks (test day 2), and after 8 weeks (test day 3) of treatment. A total intervention period of 8 weeks was chosen as this is in line with previous human intervention studies showing a significant improvement in blood lipid profiles after OLE supplementation and it has been suggested to be of sufficient duration to show a sustained effect on blood lipid levels as mentioned by the European Food Safety Authority [24, 25, 28]. The test day at 4 weeks was added to also assess the short-term effects of OLE supplementation on blood lipids. Before each test day, subjects were instructed to refrain from eating and drinking (except for water) after 10 pm in the evening. In addition, subjects were asked not to consume any alcohol-containing beverages and abstain from vigorous physical exercise from 2 days prior to testing. To limit the influence of diet, participants were asked to eat the same type of meal before each test day. Furthermore, subjects were instructed not to consume any foods containing olive phenolics during the entire duration of the study and to maintain their habitual diet. All measurements were performed in the morning in a quiet, temperature-controlled room (20–24 °C). After an overnight fast, subjects arrived at the study site, where they handed in a 3-day dietary record. Next, anthropometric (height, weight, and waist and hip circumference) and blood pressure measurements were performed. Then, blood samples were collected from an antecubital vein in the forearm and, finally, a questionnaire was completed to assess gastrointestinal symptoms, stool consistency, and stool frequency. Supplementation with the study product started after completion of the baseline measurements.

The primary objective of this study was to assess the effect of daily OLE supplementation on blood lipid profiles as measured by serum levels of total cholesterol, high-density lipoprotein (HDL)-cholesterol, low-density lipoprotein (LDL)-cholesterol, and triglycerides. The secondary objective was to assess the effect of OLE supplementation on risk markers related to the development of CVD, which were lipid peroxidation as measured by plasma-oxidized LDL (oxLDL) and blood pressure. As explorative objectives, effects on glucose metabolism and liver function parameters were assessed.

### Study product

The test product that was used was an extract prepared from olive leaves (*Olea* *europaea* L.) using a 100% water-based extraction method, standardized for its oleuropein content (> 16%), supplied by Interquim SA (Murcia, Spain). The batch used in the current study had an oleuropein content of 16.7%, providing 83.5 mg oleuropein per day. This dose was chosen based on previous studies showing a positive effect on blood lipid profiles after intake of OLEs [24–26]. As placebo, maltodextrin (Gonmisol, Barcelona, Spain) was used. The study product was provided as capsules, containing 350 mg of maltodextrin for the placebo or 250 mg of OLE in combination with 100 mg of maltodextrin. The extra 100 mg of maltodextrin was added to meet the minimum volume requirement of the capsules. Participants were asked to ingest two capsules each morning with 200 mL water 30 min before breakfast. The participants were instructed to return empty packages and unused study product during the last visit for compliance assessment.

### Blood lipids, glucose, insulin, and liver function parameters

Serum levels of total cholesterol, LDL-cholesterol, HDL-cholesterol, triglycerides, alkaline phosphatase (ALP), gamma-glutamyl transferase (GGT), aspartate aminotransferase (AST), alanine aminotransferase (ALT), total bilirubin, and plasma levels of glucose were measured by spectrophotometry (Cobas 8000 analyzer series, Roche Diagnostics, Mannheim, Germany). Serum levels of insulin were determined using an immunometric assay (XPi instrument, Siemens Medical Solutions Diagnostics, LA, USA). Blood lipids, glucose, and insulin levels were determined at baseline, after 4 weeks, and after 8 weeks of supplementation. Liver function parameters were determined at baseline and after 8 weeks.

For analysis of oxLDL, 8 mL blood was collected in an EDTA tube at baseline, after 4 weeks, and after 8 weeks of supplementation. Samples were centrifuged within 30 min at 4 °C at 1250*g* for 10 min to obtain plasma, which was stored at − 80 °C until further analysis. OxLDL measurements were performed by a sandwich ELISA procedure according to the manufacturer’s instructions (oxLDL, Mercodia AB, Uppsala, Sweden).

### Blood pressure and heart rate

Blood pressure and heart rate were assessed using a semi-continuous blood pressure monitoring device (Omron, Hoofddorp, The Netherlands) at the upper left arm after a 30-min rest in supine position. Four measurements were conducted at 5-min intervals. The first measurement was not used; the three other ones were averaged.

### Dietary intake

To assess dietary intake, participants were asked to complete a 3-day dietary record before each test day. Volunteers were asked to record the intake of 2 week days and 1 weekend day. Before the start of the study, participants were instructed on how to record their food and beverage intake, i.e*.*, based on standard household units. During each test day, the records were checked, discussed with the participant, and completed in case of missing data. Energy and nutrient intake were analyzed using the online dietary assessment tool of The Netherlands Nutrition Centre (www.voedingcentrum.nl), which is based on the Dutch Food Composition Dataset (NEVO, National Institute for Public Health and Environment, Ministry of Health, Welfare and Sport, the Hague, The Netherlands).

### Gastrointestinal tolerance, stool consistency, and stool frequency

Gastrointestinal tolerance was assessed using the gastrointestinal symptom rating scale (GSRS) as described previously [29]. To assess defecation frequency and stool consistency, the Bristol Stool Form Chart was used [30].

### Statistical analysis

The sample size calculation was determined for the primary outcome of the study, which was the effect of 8 weeks of OLE supplementation on blood lipid profiles. Based on a previous study in participants with normal cholesterol levels [25] and the assumption that a 5% stronger effect would be observed in the current study population, a sample size of at least 36 participants per group was required. A significance level of alpha = 0.05, a power of 80%, a difference in LDL-cholesterol of 0.4 mmol/L, and a standard deviation of 0.6 mmol/L were assumed for this calculation.

Statistical analyses were performed using IBM SPSS Statistics for Windows (version 25; IBM Corporation, Armonk, NY, USA). Baseline characteristics are presented as mean ± standard deviation (SD) for numerical variables and numbers for categorical variables. Analyses were performed on an intention to treat basis, including all subjects with baseline cholesterol levels between 5.0 and 8.0 mmol/L. Differences in blood lipid profiles, lipid peroxidation, blood pressure, heart rate, glucose metabolism, liver function parameters, anthropometrics, dietary intake, intestinal symptoms, stool consistency, and stool frequency were assessed by linear mixed model analyses (marginal model) with intervention group (OLE or placebo), time (baseline, 4 and 8 weeks), and intervention*time as fixed factors. For this model, an unstructured covariance structure for repeated measures was used. Results obtained with this model are presented as estimated mean ± standard error of the mean (SEM), with *p* values for the differences in means between groups after 4 and 8 weeks adjusted for baseline differences, where a two-sided *p* value ≤ 0.05 was considered statistically significant. For all outcomes, correction for multiple testing was performed by the false-discovery rate (FDR) of Benjamini–Hochberg based on a correction for multiple time points and parameters.

## Results

### Subjects

In total, 77 healthy volunteers with mildly elevated cholesterol levels were included in the study, of which 74 completed the study protocol. Two participants dropped out after completing the baseline measurements, of which one due to the need to start with medication for treatment of arrhythmia and one person due to personal circumstances. One other participant dropped out after the second test day as a precaution because of a suspected olive allergy. Baseline characteristics of the study population are presented in Table 1. Based on capsule counts, overall compliance was 97.4%.

**Table 1:** Baseline characteristics

	Total population	Placebo	OLE
	(*n* = 77)	(*n* = 38)	(*n* = 39)
Age (years)	56 ± 10	57 ± 9	56 ± 11
Male/female, *n*	35/42	19/19	16/23
Weight (kg)	85.1 ± 11.8	86.5 ± 13.3	83.8 ± 10.3
BMI (kg/m^2^)	29.0 ± 2.7	29.6 ± 2.8	28.5 ± 2.6
WHR	0.94 ± 0.08	0.95 ± 0.08	0.92 ± 0.08
Total cholesterol (mmol/L)	5.9 ± 0.7	5.7 ± 0.7	6.1 ± 0.7

All values are presented as mean ± SD or as number of participants

*BMI* body mass index, *WHR* waist-to-hip ratio, *OLE* olive leaf extract

### Blood lipid profiles

As shown in Table 2, no significant differences in changes in blood lipid profiles were observed after OLE treatment for 8 weeks compared to placebo. After 4 weeks, there were a significant decrease in triglyceride levels (*p* = 0.028) and a significant improvement in the triglyceride-to-HDL-cholesterol ratio in the OLE group compared to the placebo (*p* = 0.018), but these effects did not remain significant after correction for multiple testing (both p > 0.05). Furthermore, OLE supplementation did not significantly affect oxLDL levels compared to placebo (Table 2).

**Table 2:** Blood lipid and oxLDL concentrations at baseline, after 4 weeks, and after 8 weeks of supplementation

	Placebo			OLE			Uncorrected *p*_1_	Uncorrected *p*_2_
	Baseline	4 weeks	8 weeks	Baseline	4 weeks	8 weeks		
Total cholesterol (mmol/L)	5.8 ± 0.1	5.7 ± 0.1	5.7 ± 0.1	6.1 ± 0.1	6.0 ± 0.1	6.0 ± 0.1	0.472	0.838
HDL-cholesterol (mmol/L)	1.6 ± 0.1	1.6 ± 0.1	1.6 ± 0.1	1.7 ± 0.1	1.7 ± 0.1	1.6 ± 0.1	0.575	0.490
Non-HDL-cholesterol (mmol/L)	4.1 ± 0.1	4.1 ± 0.1	4.0 ± 0.1	4.4 ± 0.1	4.3 ± 0.1	4.3 ± 0.1	0.586	0.960
LDL-cholesterol (mmol/L)	3.6 ± 0.1	3.5 ± 0.1	3.5 ± 0.1	3.8 ± 0.1	3.7 ± 0.1	3.8 ± 0.1	0.802	0.571
Triglycerides (mmol/L)	1.2 ± 0.1	1.3 ± 0.1	1.3 ± 0.1	1.3 ± 0.1	1.2 ± 0.1	1.2 ± 0.1	0.028^a^	0.176
Total cholesterol/HDL-cholesterol	3.8 ± 0.2	3.8 ± 0.2	3.7 ± 0.2	3.9 ± 0.2	3.8 ± 0.2	3.9 ± 0.2	0.501	0.852
Triglycerides/HDL-cholesterol	0.8 ± 0.1	0.9 ± 0.1	0.9 ± 0.1	0.9 ± 0.1	0.8 ± 0.1	0.9 ± 0.1	0.018^a^	0.155
OxLDL (U/L)	72.8 ± 2.8	73.1 ± 2.9	73.9 ± 3.1	83.6 ± 2.9	82.3 ± 2.9	83.3 ± 3.0	0.595	0.613

All values are presented as mean ± SEM. Differences between the placebo and OLE were compared with an unstructured linear mixed model with correction for baseline values. *p*_1_ and *p*_2_ represent the *p* values for the difference in estimated means after 4 and 8 weeks of intervention, respectively, between placebo and OLE, corrected for baseline differences

*HDL* high-density lipoprotein, *LDL* low-density lipoprotein, *OxLDL* oxidized low-density lipoprotein, *OLE* olive leaf extract

^a^*p* > 0.05 after correction for multiple testing by the false-discovery rate (FDR) of Benjamini–Hochberg (16 tests)

### Hemodynamic parameters

Systolic blood pressure, mean arterial pressure, pulse pressure, and heart rate were not significantly altered after 4 weeks or 8 weeks of supplementation with OLE compared to placebo (all *p* ≥ 0.095; Table 3). Diastolic blood pressure showed a slight decrease in the placebo group after 4 weeks of supplementation compared to OLE (*p* = 0.029), but this effect was not significant after correction for multiple testing, nor after 8 weeks of intake.

**Table 3:** Hemodynamic parameters at baseline, after 4 weeks, and after 8 weeks of supplementation

	Placebo			OLE			Uncorrected *p*_1_	Uncorrected *p*_2_
	Baseline	4 weeks	8 weeks	Baseline	4 weeks	8 weeks		
Systolic BP (mmHg)	131 ± 2	129 ± 2	131 ± 2	128 ± 2	128 ± 2	127 ± 2	0.501	0.731
Diastolic BP (mmHg)	82 ± 2	81 ± 2	82 ± 1	80 ± 2	80 ± 2	79 ± 1	0.029^a^	0.590
MAP (mmHg)	99 ± 2	97 ± 2	98 ± 2	96 ± 2	96 ± 2	95 ± 2	0.095	0.905
Pulse pressure (mmHg)	49 ± 1	49 ± 2	49 ± 1	49 ± 1	48 ± 1	48 ± 1	0.473	0.428
HR (bpm)	66 ± 2	67 ± 2	68 ± 2	67 ± 2	67 ± 2	67 ± 2	0.803	0.532

All values are presented as mean ± SEM. Differences between the placebo and OLE were compared with an unstructured linear mixed model with correction for baseline values. *p*_1_ and *p*_2_ represent the *p* values for the difference in estimated means after 4 and 8 weeks of intervention, respectively, between placebo and OLE, corrected for baseline differences

*BP* blood pressure, *MAP* mean arterial pressure, *HR* heart rate, *OLE* olive leaf extract

^a^*p* > 0.05 after correction for multiple testing by the false-discovery rate (FDR) of Benjamini–Hochberg (10 tests)

### Glucose and insulin

At baseline, blood glucose and insulin levels were both within normal blood value ranges (Table 4). Although glucose levels showed a slight but significant increase after 8 weeks of supplementation compared to placebo (*p* = 0.024), this effect did not remain significant after correction for multiple testing. No significant difference in changes was observed for glucose levels after 4 weeks or for insulin levels throughout the study as a result of OLE supplementation compared to placebo (all *p* ≥ 0.155).

**Table 4:** Glucose and insulin concentrations at baseline, after 4 weeks, and after 8 weeks of supplementation

	Placebo			OLE			Uncorrected *p*_1_	Uncorrected *p*_2_
	Baseline	4 weeks	8 weeks	Baseline	4 weeks	8 weeks		
Glucose (mmol/L)	5.2 ± 0.1	5.2 ± 0.1	5.2 ± 0.1	5.2 ± 0.1	5.3 ± 0.1	5.3 ± 0.1	0.229	0.024^a^
Insulin (pmol/L)	87.7 ± 21.0	97.7 ± 23.6	92.7 ± 24.8	65.1 ± 20.7	55.1 ± 23.2	62.5 ± 24.5	0.155	0.467

All values are presented as mean ± SEM. Differences between the placebo and OLE were compared with an unstructured linear mixed model with correction for baseline values. *p*_1_ and *p*_2_ represent the *p* values for the difference in estimated means after 4 and 8 weeks of intervention, respectively, between placebo and OLE, corrected for baseline differences

*OLE* olive leaf extract

^a^*p* > 0.05 after correction for multiple testing by the false-discovery rate (FDR) of Benjamini–Hochberg (4 tests)

### Anthropometrics and dietary intake

Anthropometrics and dietary intake were monitored during the study period to check whether participants maintained their dietary habits during the study period. No significant differences in body weight, BMI, waist-to-hip ratio (WHR), or dietary intake were observed between both interventions over time (Table 5).

**Table 5:** Anthropometrics and dietary intake at baseline, after 4 weeks, and after 8 weeks of supplementation

	Placebo			OLE			Uncorrected *p*_1_	Uncorrected *p*_2_
	Baseline	4 weeks	8 weeks	Baseline	4 weeks	8 weeks		
Weight (kg)	86.5 ± 1.9	86.6 ± 1.9	86.6 ± 1.9	83.8 ± 1.9	83.6 ± 1.9	83.9 ± 1.9	0.308	0.873
BMI (kg/m^2^)	29.5 ± 0.4	29.5 ± 0.5	29.5 ± 0.4	28.5 ± 0.4	28.5 ± 0.4	28.6 ± 0.4	0.451	0.839
WHR	0.95 ± 0.01	0.94 ± 0.01	0.95 ± 0.01	0.92 ± 0.01	0.92 ± 0.01	0.92 ± 0.01	0.768	0.884
Energy (kcal)	2022 ± 65	1990 ± 84	1977 ± 74	1977 ± 64	2024 ± 81	1981 ± 72	0.490	0.613
Fat (g)	78.6 ± 3.6	78.3 ± 4.9	80.6 ± 4.0	79.5 ± 3.5	84.3 ± 4.7	82.3 ± 3.9	0.475	0.891
Saturated fat (g)	28.5 ± 1.6	29.2 ± 1.6	28.6 ± 1.8	28.2 ± 1.6	30.2 ± 1.6	30.4 ± 1.7	0.603	0.430
Carbohydrates (g)	219.3 ± 8.6	212.6 ± 11.1	206.0 ± 9.7	209.0 ± 8.4	215.3 ± 10.7	203.4 ± 9.5	0.425	0.525
Fiber (g)	21.3 ± 1.2	20.8 ± 1.2	20.2 ± 1.2	20.3 ± 1.2	20.7 ± 1.2	20.2 ± 1.1	0.395	0.398
Protein (g)	83.1 ± 3.5	85.1 ± 3.7	81.8 ± 3.5	80.6 ± 3.5	85.4 ± 3.6	84.9 ± 3.4	0.629	0.238
Salt (g)	6.9 ± 0.6	6.8 ± 1.2	6.6 ± 0.5	6.7 ± 0.6	8.8 ± 1.1	6.8 ± 0.4	0.231	0.580
Alcohol (g)	6.7 ± 1.7	5.2 ± 1.4	5.4 ± 1.5	9.3 ± 1.7	8.2 ± 1.4	6.7 ± 1.5	0.899	0.420

All values are presented as mean ± SEM. Differences between the placebo and OLE were compared with an unstructured linear mixed model with correction for baseline values. *p*_1_ and *p*_2_ represent the *p* values for the difference in estimated means after 4 and 8 weeks of intervention, respectively, between placebo and OLE, corrected for baseline differences

*BMI* body mass index, *WHR* waist-to-hip ratio, *OLE* olive leaf extract

### Liver function parameters

Serum ALP, GGT, AST, ALT, and bilirubin were all within normal blood value ranges throughout the study period (Supplemental Table 1) and were not significantly affected by OLE supplementation compared to placebo.

### Gastrointestinal tolerance, stool frequency, and stool consistency

Gastrointestinal tolerance, as assessed by GSRS sub-dimension scores (i.e., abdominal pain, reflux, diarrhea, indigestion, and constipation), did not show a significant difference in change from baseline throughout the study period between groups (Supplemental Table 2). Furthermore, no significant difference in changes in stool frequency or stool consistency was observed between OLE and placebo treatment (Supplemental Table 2).

## Discussion

In this study, the effect of 8 weeks of OLE supplementation on markers of CVD was investigated in overweight/obese participants with mildly elevated cholesterol levels. OLE supplementation did not significantly affect blood lipid levels after 4 weeks or after 8 weeks compared to placebo. Furthermore, no statistically significant differences in oxLDL, blood pressure, glucose, and insulin levels or liver function parameters were found between the two intervention groups.

Increased levels of total cholesterol and LDL-cholesterol, and decreased levels of HDL-cholesterol are well-known CVD risk factors [4, 31]. In addition, evidence also indicates that elevated triglyceride levels and in particular an elevated triglyceride to HDL-cholesterol ratio might be a useful marker for CVD risk, as it has been associated with plasma levels of small dense LDL particles [32, 33]. In the current study, no significant effects of OLE (containing 83.5 mg oleuropein) on lipid levels were found after 8 weeks of supplementation. Our findings are in line with findings by de Bock et al*.* [23]. In their study, daily supplementation with OLE (containing 51.1 mg oleuropein and 9.7 mg hydroxytyrosol) for 12 weeks in overweight middle-aged men resulted in an improved insulin sensitivity, but had no significant effect on blood lipid profiles. Other studies, however, have found improved lipid profiles after OLE supplementation, although the outcomes and their effect sizes differ between studies. In pre-hypertensive males, a significant decrease in total cholesterol, LDL-cholesterol, and triglyceride levels was observed after OLE (containing 136 mg oleuropein and 6 mg hydroxytyrosol) intake for 6 weeks [24]. Supplementation with OLE containing 200 mg oleuropein for 8 weeks in patients with stage-1 hypertension also resulted in reduced total cholesterol, LDL-cholesterol, and triglyceride levels [27]. A decrease in LDL-cholesterol was shown after 8 weeks of supplementation with OLE at dosages containing 104 and 208 mg oleuropein in monozygotic twins with mild hypertension [25]. In all three of these OLE intervention studies, participants were selected based on their blood pressure levels, participants had slightly lower cholesterol values compared to the current study population, and different control conditions were used. These control conditions consisted of a liquid formula control designed to match the liquid OLE product in appearance, taste, texture, and aroma as closely as possible [24], an active control (Captopril) [27], and lifestyle advice [25]. Furthermore, higher dosages of olive phenolics (104–208 mg vs 83.5 mg daily) were used, although the actual dose may have been lower than that in some cases [24]. Improvements in blood lipids have been demonstrated at much lower phenolic dosages (~12.5 and 9 mg per day) and after a much shorter interval of only 3 weeks of supplementation with olive oils rich in polyphenols in healthy subjects [34] and in subjects with hypercholesterolemia [35]. In both trials [34, 35], a significant decrease in LDL-cholesterol concentrations and various other atherogenic markers were found with olive oil rich in polyphenols compared to olive oil low in polyphenol levels but with identical fat and micronutrient composition. It is, however, remarkable that these two olive oil studies were able to show a decrease in blood lipids after supplementation at much lower phenolic dosages as compared to the current study. A clear explanation is lacking, though differences in study design should be noted. For example, olive oil and olive leaf differ in phenolic composition, with olive leaves containing significantly higher concentrations of oleuropein and glycosylated flavones, while olive oil is generally higher in flavone aglycones [13]. Furthermore, we cannot exclude that differences in factors that can influence phenolic bioavailability such as food matrix, gender, and microbiome perturbations associated with overweight and obesity have played a role [13, 36, 37]. In post-menopausal women with osteopenia, OLE supplementation containing approximately 100 mg oleuropein resulted in a significant improvement of total cholesterol, LDL-cholesterol, and triglyceride levels after 12 months [26]. In that specific trial, the primary focus was on bone health, which is why a study duration of 12 months was chosen. We have chosen a study duration of 8 weeks, in line with most previous OLE trials. We also assessed the effects after 4 weeks, because if short-term beneficial effects can be shown, this could positively affect patient’s compliance for potential future applications. Furthermore, a functional food instead of dietary intervention was chosen, as long-term adherence to dietary regimens is often poor [38].

As previous studies have shown that certain diets or dietary components such as trans-fatty acids can affect lipid levels, e.g., LDL-cholesterol, HDL-cholesterol, and triglycerides [39–41], participants were instructed to consume similar meals the night before each test day and to maintain their dietary habits throughout the entire study period. It is therefore unlikely that the results of the current study were influenced by the diet of the participants. Data from the anthropometric measurements and the food records do corroborate this, as there were no significant changes over time between the intervention groups with regard to body weight, WHR, and dietary intake.

In close relation to dyslipidemia, hypertension and oxLDL are important risk factors for the development of atherosclerosis and CVD [3, 42]. Previous studies have shown improvements in systolic and diastolic blood pressure after intake of OLEs with a phenolic content of ~ 100–200 mg per day for 6 or 8 weeks, using both 24-h ambulatory blood pressure and blood pressure measurements at a single time point [24, 25, 27]. These studies all have in common that the participants had elevated blood pressure levels. One study that did not include participants based on blood pressure levels failed to show significant effects on systolic or diastolic blood pressure after 12 weeks of intake [23]. In the current study, we also did not observe a significant effect of OLE supplementation on systolic or diastolic blood pressure compared to the placebo, nor on oxLDL levels.

Elevated glucose and/or insulin levels and altered liver function parameters are associated with overweight and obesity [2, 43]. As mentioned previously, an improvement in insulin sensitivity as a result of 12 weeks of OLE supplementation has been shown in middle-aged overweight men [23]. Using an oral glucose tolerance test, a reduction in the area under the curve for both glucose and insulin was observed. Furthermore, supplementation with 500 mg OLE for 14 weeks in patients with type 2 diabetes resulted in significantly lower HbA1c levels [20]. We did not find any significant improvements in either glucose or insulin levels after intake of OLE. When taking into account the baseline values within our population, these findings were to be expected, as glucose and insulin levels were all within normal ranges and our study design did not include a glucose challenge. Similarly, no significant effects on liver function parameters were observed.

Gastrointestinal symptom scores were assessed to monitor gastrointestinal tolerance during the supplementation period. OLE had no significant impact on gastrointestinal symptom scores, indicating that the study product was well-tolerated. Furthermore, with the exception of one participant (allergic symptoms), no adverse events were reported as a result of the intake of the study product.

Some limitations of the current study have to be addressed. First, for several outcome measures, there were slight differences in baseline values between the two intervention groups. However, these differences did not have an impact on our conclusions as statistical analyses were performed with correction for baseline values. Second, the changes in blood lipids, blood pressure, and glucose levels were very small, and do not appear to be clinically relevant. Third, based on the dietary food records, it was not possible to calculate the intake of trans-fatty acids within our study population. However, as there were no significant changes in the intake of the major food components over time, we do not expect that the intake of trans-fatty acids could have affected our outcomes. Fourth, the dose that was used in the current study was chosen based on a previous study using an OLE at a dose of approximately 100 mg oleuropein, which showed an effect after 12 months of supplementation [26]. We aimed to confirm these longer term effects on blood lipid profiles at this dose of oleuropein in a relevant target population during a much shorter lasting supplementation. It should be taken into account that due to differences in the extraction method applied (water vs ethanol/water) and the resulting oleuropein content between extracts, the final dose of the current study product was 83.5 mg per day instead of 100 mg per day in the long-term supplementation study [26]. In previous studies of similar or shorter duration, positive effects on blood lipids have been shown for OLE at daily dosages of approximately 100 mg of oleuropein or even lower dosages for olive oil phenolics [25, 34, 35]. In our study, all subjects were selected based on mildly elevated baseline total cholesterol levels. In patients with higher total cholesterol levels, outcomes might have been different, but in these patients, cholesterol-lowering medication is indicated.

In conclusion, we have shown that OLE supplementation at a dose of 83.5 mg OLE phenolics per day for 8 weeks in overweight/obese participants with mildly elevated cholesterol had no significant effect on blood lipid profiles as primary parameter or any of the other markers related to CVD as secondary parameters.

## Electronic supplementary material

Below is the link to the electronic supplementary material.

Supplementary file1 (PDF 57 kb)
