# Supplementary material for: The effect of olive leaf extract on cardiovascular health markers: a randomized placebo-controlled clinical trial
Source: Eur J Nutr. 2020 Oct 9;60(4):2111–20. doi: 10.1007/s00394-020-02397-9 (PMC8137474; doi:10.1007/s00394-020-02397-9)
Supplement: Supplementary file 1 — Supplementary file1 (PDF 57 kb) [file 394_2020_2397_MOESM1_ESM.pdf]

THE EFFECT OF OLIVE LEAF EXTRACT ON CARDIOVASCULAR HEALTH MARKERS: A RANDOMIZED PLACEBO-CONTROLLED CLINICAL TRIAL

European Journal of Nutrition

Yala Stevens[1, 2], Bjorn Winkens[3], Daisy Jonkers[1], Adrian Masclee[1]

[1] Department of Internal Medicine, Division of Gastroenterology-Hepatology, School of Nutrition and Translational Research in Metabolism (NUTRIM), Maastricht University, Maastricht, The Netherlands

[2] BioActor BV, Maastricht, The Netherlands

[3] Department of Methodology and Statistics, Care and Public Health Research Institute (CAPHRI), Maastricht University, Maastricht, the Netherlands.

Corresponding author

Yala Stevens

E-mail: yala.stevens@maastrichtuniversity.nl

SUPPLEMENTAL TABLES

Supplemental Table 1. Liver function parameters at baseline and after 8 weeks of supplementation

	Placebo		OLE		*Uncorrected* *P* value
	Baseline	8 weeks	Baseline	8 weeks	
ALP (U/l)	72.9 ± 3.0	72.5 ± 3.3	77.0 ± 2.9	78.1 ± 3.2	0.425
GGT (U/l)	32.6 ± 3.8	32.0 ± 3.8	25.1 ± 3.7	25.1 ± 3.8	0.792
AST (U/l)	23.8 ± 1.1	22.4 ± 1.4	23.7 ± 1.1	23.7 ± 1.4	0.320
ALT (U/l)	27.3 ± 2.1	26.7 ± 2.9	27.1 ± 2.1	29.3 ± 2.9	0.246
Bilirubin (μmol/l)	10.3 ± 1.0	11.6 ± 1.1	9.0 ± 1.0	9.6 ± 1.1	0.600

All values are presented as mean ± SEM. Differences between the placebo and OLE after 8 weeks of supplementation were compared with an unstructured linear mixed model with correction for baseline values. ALP, alkaline phosphatase; GGT, gamma-glutamyl transferase; AST, aspartate aminotransferase; ALT, alanine aminotransferase.

Supplemental Table 2. GSRS sub scores, stool frequency and stool consistency at baseline, after 4 weeks and after 8 weeks of supplementation

	Placebo			OLE			*Uncorrected* P_1	*Uncorrected* P_2
	Baseline	4 weeks	8 weeks	Baseline	4 weeks	8 weeks		
Abdominal pain	1.4 ± 0.1	1.4 ± 0.1	1.4 ± 0.1	1.5 ± 0.1	1.4 ± 0.1	1.5 ± 0.1	0.941	0.768
Reflux	1.3 ± 0.1	1.2 ± 0.1	1.2 ± 0.1	1.3 ± 0.1	1.3 ± 0.1	1.4 ± 0.1	0.750	0.215
Diarrhea	1.4 ± 0.1	1.7 ± 0.1	1.5 ± 0.1	1.4 ± 0.1	1.6 ± 0.1	1.7 ± 0.1	0.589	0.379
Indigestion	1.9 ± 0.1	1.6 ± 0.1	1.7 ± 0.1	1.9 ± 0.1	1.8 ± 0.1	1.9 ± 0.1	0.137	0.202
Constipation	1.5 ± 0.1	1.5 ± 0.1	1.6 ± 0.1	1.4 ± 0.1	1.4 ± 0.1	1.5 ± 0.1	0.798	0.652
Stool frequency	1.6 ± 0.2	1.7 ± 0.1	1.7 ± 0.1	1.5 ± 0.2	1.4 ± 0.1	1.3 ± 0.1	0.195	0.160
Stool type[a]	3.7 ± 0.2	3.8 ± 0.2	3.8 ± 0.2	3.5 ± 0.2	3.7 ± 0.2	3.6 ± 0.2	0.557	0.879

All values are presented as mean ± SEM. Differences between the placebo and OLE were compared with an unstructured linear mixed model with correction for baseline values. P_1 and P_2 represent the *P* values for the difference in estimated means after 4 and 8 weeks of intervention, respectively, between placebo and OLE, corrected for baseline differences. GSRS, gastrointestinal symptom rating scale. [a] Values represent average self-reported scores over a period of 1 week.

SUPPLEMENTAL FIGURES

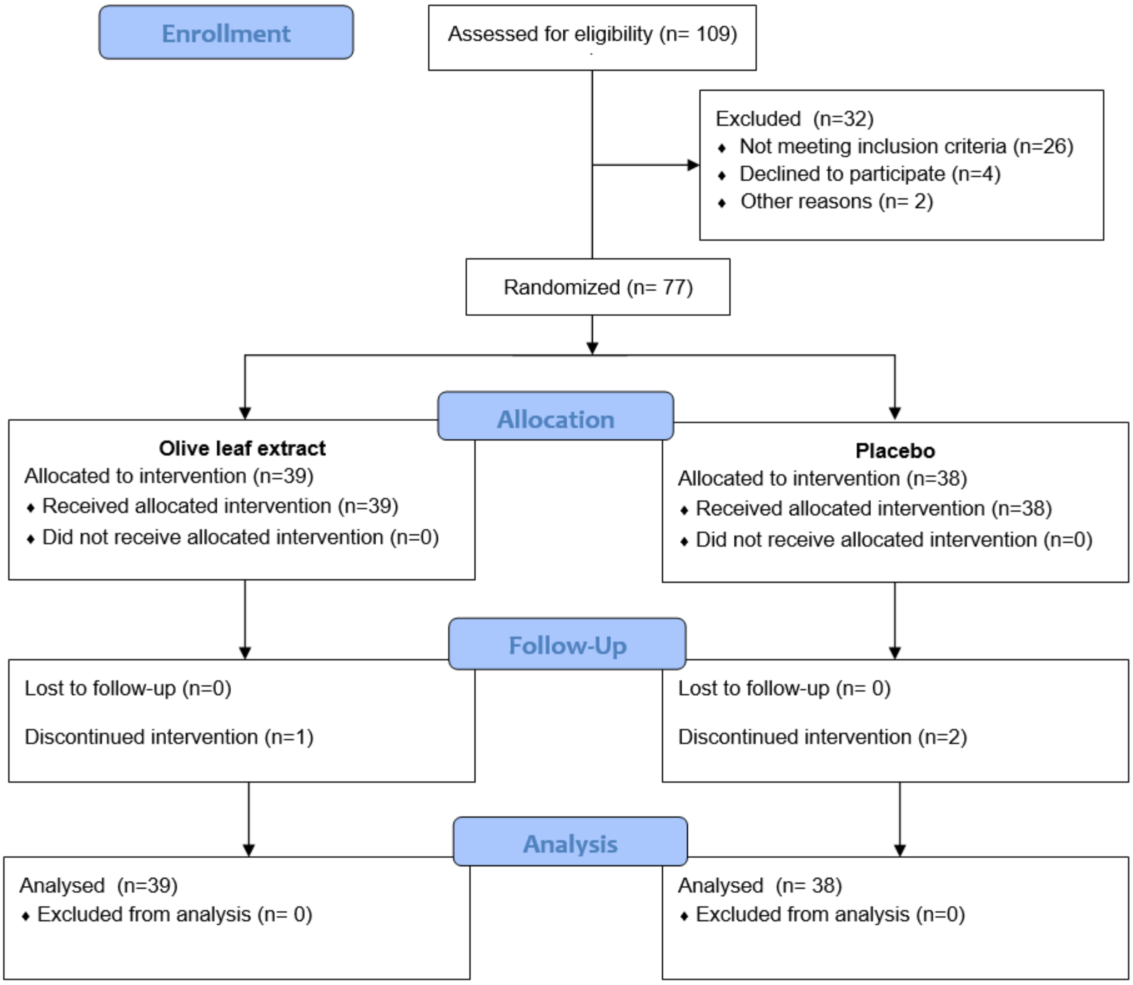

Supplemental Figure 1. CONSORT flow diagram
